# Therapeutic Aspects of *Prunus cerasus* Extract in a Rabbit Model of Atherosclerosis-Associated Diastolic Dysfunction

**DOI:** 10.3390/ijms241713253

**Published:** 2023-08-26

**Authors:** Reka Szekeres, Daniel Priksz, Rita Kiss, Dana Diana Romanescu, Mariann Bombicz, Balazs Varga, Rudolf Gesztelyi, Anna Szilagyi, Barbara Takacs, Vera Tarjanyi, Beata Pelles-Tasko, Ildiko Forgacs, Judit Remenyik, Zoltan Szilvassy, Bela Juhasz

**Affiliations:** 1 Department of Pharmacology and Pharmacotherapy, Faculty of General Medicine, University of Debrecen, H-4032 Debrecen, Hungary; szekeres.reka@med.unideb.hu (R.S.); priksz.daniel@pharm.unideb.hu (D.P.); kiss.rita@med.unideb.hu (R.K.); bombicz.mariann@pharm.unideb.hu (M.B.); varga.balazs@pharm.unideb.hu (B.V.); gesztelyi.rudolf@pharm.unideb.hu (R.G.); dr.szilagyi.anna@med.unideb.hu (A.S.); takacs.barbara@pharm.unideb.hu (B.T.); tarjanyi.vera@med.unideb.hu (V.T.); pelles-tasko.beata@med.unideb.hu (B.P.-T.); szilvassy.zoltan@med.unideb.hu (Z.S.); 2 Department of Diabetology, Pelican Clinical Hospital, 410087 Oradea, Romania; danamed2000@yahoo.com; 3 Department of Medical Disciplines, Faculty of Medicine and Pharmacy, University of Oradea, 410087 Oradea, Romania; 4 Center for Complex Systems and Microbiome Innovations, Faculty of Agricultural and Food Sciences and Environmental Management, University of Debrecen, H-4032 Debrecen, Hungary; forgacs.ildiko@agr.unideb.hu (I.F.); remenyik@agr.unideb.hu (J.R.)

**Keywords:** anthocyanins, cardiac dysfunction, *Prunus cerasus*, eNOS, PKG, SERCA2a, HO-1

## Abstract

This study evaluates the potential therapeutic effects of anthocyanin-rich *Prunus cerasus* (sour cherry) extract (PCE) on atherosclerosis-associated cardiac dysfunction, described by the impairment of the NO-PKG (nitric oxide–protein kinase G) pathway and the antioxidant capacity. Initially, a rabbit model of atherosclerotic cardiovascular disease was established by administering a cholesterol-rich diet, enabling the examination of the impact of 9 g/kg PCE on the pre-existing compromised cardiovascular condition. After that, the animals were divided into four groups for 12 weeks: the (1) untreated control group; (2) PCE-administered healthy rabbits; (3) hypercholesterolemic (HC) group kept on an atherogenic diet; and (4) PCE-treated HC group. Dyslipidemia, impaired endothelial function, and signs of diastolic dysfunction were evident in hypercholesterolemic rabbits, accompanied by a reduced cardiac expression of eNOS (endothelial nitric oxide synthase), PKG, and SERCA2a (sarco/endoplasmic reticulum calcium ATPase 2a). Subsequent PCE treatment improved the lipid profile and the cardiac function. Additionally, PCE administration was associated with elevated myocardial levels of eNOS, PKG, and SERCA2a, while no significant changes in the vascular status were observed. Western blot analysis further revealed hypercholesterolemia-induced increase and PCE-associated reduction in heme oxygenase-1 expression. The observed effects of anthocyanins indicate their potential as a valuable addition to the treatment regimen for atherosclerosis-associated cardiac dysfunction.

## 1. Introduction

Despite the improving the treatment of cardiovascular diseases, the overall incidence of heart failure (HF) is still increasing [1,2,3]. Coronary artery disease (CAD), hypertension, and diabetes mellitus are the predominant risk factors for HF in developed and Western-type countries [4,5]. HF itself is the clinical consequence, whereas cardiac (systolic or diastolic) dysfunction, as a precursor of the condition, refers to the underlying functional changes that are present before the onset of the symptoms of the disease. The prognosis of the condition remains poor; moreover, the quality of life is also diminished, suggesting the fact that expanding the possibilities of the pharmacological treatment of HF—especially diastolic dysfunction—is essential in the current research on cardiovascular diseases. 

Patients with diastolic dysfunction (DD) suffer from impaired left ventricle (LV) relaxation and filling during the cardiac cycle. These abnormalities are characterized by enhanced ventricular stiffness due to alterations in both the extracellular matrix (ECM) and the cardiomyocytes [6]. Evidence suggests that the nitric oxide (NO)–cyclic guanosine monophosphate (cGMP)–protein kinase G (PKG) signalization cascade plays a crucial role in cardiovascular physiology [7]. NO, the main molecule responsible for the maintenance of vascular integrity, is synthesized by endothelial NO synthase (eNOS) under the stimulation of physical or chemical factors [8]. NO may diffuse to the cardiomyocytes surrounding the coronary capillaries, and in the myocardium, it increases the intracellular concentrations of cGMP by activating soluble guanylate cyclase (sGC). The most important downstream target of cGMP is PKG, which regulates several molecular signal transduction pathways [9]. The proper relaxation of the myocardium is provided by the downstream signaling targets of the NO-cGMP-PKG pathway controlling calcium (Ca^2+^) homeostasis with the help of the sarcoplasmic reticulum Ca^2+^-APTase (SERCA2a), as well as regulating the giant elastic protein called titin [10].

Metabolic comorbidities such as diet-induced hyperlipidemia and obesity are important contributors to atherosclerotic cardiovascular diseases, in which oxidative stress and inflammatory processes cause endothelial dysfunction observed also in the coronary microvasculature and affect NO bioavailability [11]. Thus, downregulated NO-cGMP-PKG paracrine signaling develops cardiomyocyte hypertrophy and high resting tension [12]. A large number of studies in the scientific literature have examined how heat shock proteins are involved in cardioprotective mechanisms. Abundant evidence indicates that the upregulation of the heme oxygenase-1 (HO-1, also known as hsp32) plays a pivotal role among the defense mechanisms activated by cells in response to oxidative stress stimuli closely associated with the atherosclerotic process [13,14]. However, the idea that high HO-1 levels are not necessarily beneficial is also gaining ground nowadays.

In recent years, anthocyanins, a subgroup of flavonoids, have garnered increasing interest in the medicinal field due to their antioxidant and anti-inflammatory properties related to their chemical structure [15]. The main sources of these bioactive compounds are flowers and fruits (e.g., red berry fruits, especially sour cherries, black raspberries, blackcurrants, and bilberries; red wine; pomegranates; and red cabbage varieties) [16,17]. The medical literature shows hard evidence of the fact that anthocyanins are promising agents in the treatment of several diseases with their cytoprotective [18], anticancer [19], antidiabetic [20], antimicrobial [21], anti-obesity [22], and anti-inflammatory [23] effects. Furthermore, anthocyanins are thought to have cardioprotective activities by ameliorating the lipid profile, inhibiting LDL oxidation, decreasing the circulating C-reactive protein (CRP) levels, and improving the endothelial function via the activation of the NO-cGMP signaling pathway [24,25]. Last, but not least, these natural compounds have been found to moderate oxidative-stress-induced injury by stimulating HO-1 signaling pathways [13,26].

The scientific literature suggests that low-dose-cholesterol-fed rabbits are valuable models of dyslipidemia, atherosclerosis, and cardiac dysfunction. These experimental animals are very susceptible to an atherogenic diet—at least in part—as this type of diet can upregulate cholesteryl ester transfer protein (CETP) expression [27,28]. Additionally, rabbits have a myocardium and cellular electrophysiology structure similar to that of humans [29].

The authors of this publication consider it important to note that in the animal model used, the disease under investigation had already been developed; thus, we were interested in therapeutic rather than preventive options. Anthocyanins are often used for preventing and delaying the onset of a disease; however, we examined them as treatment options focusing on managing and alleviating the underlying causes of the existing compromised cardiovascular condition.

The primary objective of the present study is to elucidate the therapeutic effects of anthocyanin-rich sour cherry (*Prunus cerasus* L.) extract on cardiac dysfunction in a rabbit model of atherosclerotic cardiovascular disease.

## 2. Results

### 2.1. Main Compounds of Anthocyanin-Rich PCE

**Figure 1:**
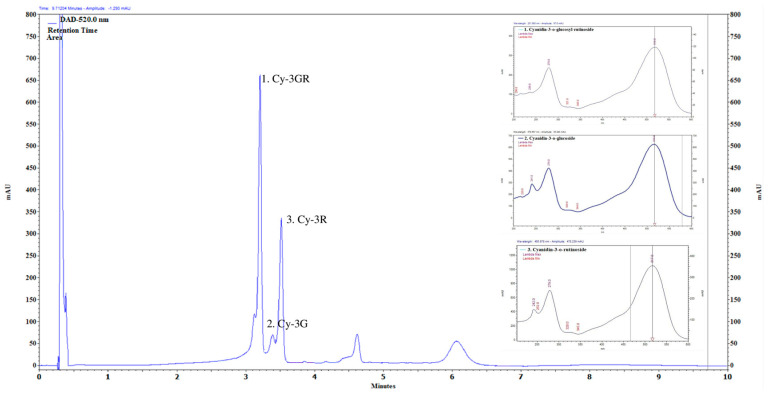
Chromatograms of the anthocyanin compounds. 1. Cyanidin-3-o-glucosyl-rutinoside (52.5 mg/100 g). 2. Cyanidin-3-o-glucoside (3.97 mg/100 g). 3. Cyanidin-3-rutinoside (7.02 mg/100 g).

The main anthocyanin components in the extract were cyanidin-3-O-rutinoside, cyanidin-3-O-glucoside, and cyanidin-3-O-glucosyl-rutinoside, and a few phenolic compounds were present in a negligible amount (Figure 1). The sample components were separated using an Aquity UPLC BEH Shield RP18 1.7 μm. UHPLC running conditions consisted in the following linear gradient steps: 0 min solvent A 99%, 5 min solvent A 75%, 5.10 min solvent A 75%, 6.50 min solvent A 0%, 7 min solvent A 0%, and 10.0 min solvent A 99%. Solvent A: ACN; solvent B: 4.5% HCOOH (formic acid) in water. Flow rate was 0.450 mL min^−1^ and oven temperature was kept at 25 ℃. The anthocyanin content was analyzed quantitatively through comparison with the corresponding authentic standards. UV–vis detection was used at a 520 nm wavelength for anthocyanins compounds. Injection volume was 5 µL.

### 2.2. PCE Treatment Moderates Diet-Induced Dyslipidemia

Evaluated lipid parameters and cardiac biomarkers are shown in Table 1. Levels of total cholesterol and LDL-C were significantly increased in the HC group compared to controls (*p* < 0.0001 in both cases). HDL-C levels showed a similar trend (control vs HC: *p* = 0.0022). Results of the atherogenic index of plasma (AIP) in rabbits from the HC group were significantly higher than those calculated in the healthy control animals (*p* < 0.0001). In HC rabbits treated with PCE (HC + PCE), a significant improvement in the lipid profile was observed compared to the HC group. The PCE administration significantly reduced the levels of both total cholesterol and LDL-C in the HC + PCE animals, versus the HC group (TC: *p* = 0.0013; LDL-C: *p* < 0.0001). Moreover, AIP values significantly decreased in the anthocyanin-treated HC rabbits, compared to the animals in the HC group (*p* = 0.0028). Additionally, no significant differences were detected in the serum triglyceride contents of the experimental groups. It was further noted that the CRP concentration was four-fold higher in the serum of the hypercholesterolemic rabbits compared to the healthy groups and four-fold lower in the HC + PCE animals, versus the non-treated HC group. No perceptible changes were identified in the cardiac biomarker CK-MB levels. Concerning glucose levels and liver enzymes (AST and ALT), we observed no marked changes. As a limitation of the study, the levels of NT-pro-BNP were below the detection limit in all treatment groups.

### 2.3. Atherosclerosis-Associated Cardiac Dysfunction Was Attenuated by Anthocyanin Administration

**Figure 2:**
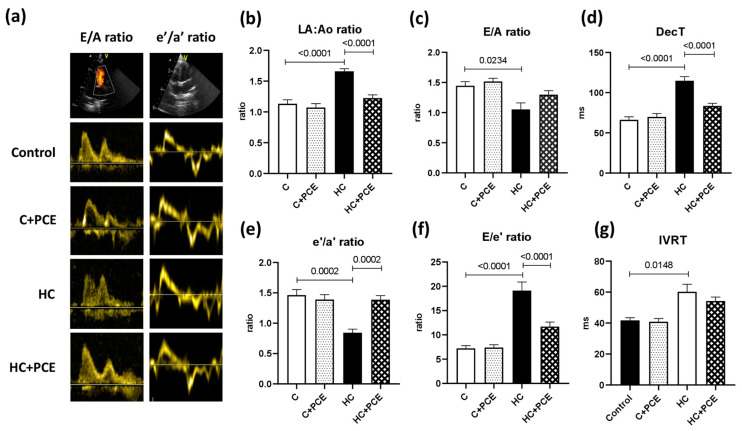
Echocardiographic parameters representing diastolic performance (f 7 in each group). (**a**) Representative Doppler patterns of the mitral inflow (E/A ratio) and representative Tissue Doppler recordings of the septal mitral annular velocities (e’/a’ ratio). (**b**) Left atrial-to-aortic root diameter (LA/Ao) ratio increased in the HC but reduced in the HC + PCE groups. (**c**) Transmitral E/A ratio worsened in the HC animals. (**d**) Deceleration time (DecT) lengthened in the HC rabbits but was restored in the HC + PCE group. (**e**) e’/a’ ratio deteriorated in the HC but improved in the HC + PCE animals. (**f**) Calculated E/e’, indicating left ventricle filling pressure was elevated in the HC but attenuated in the HC + PCE rabbits. (**g**) Isovolumetric relaxation time (IVRT) increased in the rabbits kept on an atherogenic diet. Data are presented as means ± SEM. All data followed normal distribution so they were analyzed with ordinary one-way ANOVA followed by Tukey’s post hoc test.

The results of the cardiac ultrasound are summarized in Table 2. Hypercholesterolemic (HC) rabbits showed marked signs of diastolic dysfunction compared to controls. In the HC group, left atrial-to-aortic root diameter (LA/Ao) ratio increased (*p* < 0.0001), deceleration time (DecT) and isovolumetric relaxation time (IVRT) increased (DecT: *p* <0.0001; IVRT: *p* = 0.0148), and E/A ratio and e’/a’ ratio decreased (E/A ratio: *p* = 0.0234; e’/a’ ratio: *p* = 0.0002) in comparison to healthy rabbits. The average E/e’ ratio observed in the HC animals was significantly higher compared to the control group (*p* = <0.0001), estimating an increase in the left ventricle (LV) filling pressure in the non-treated HC group. We detected a significant improvement in the echocardiographic parameters representing the diastolic function in the PCE-treated group (HC + PCE) versus the HC rabbits (Figure 2). In hypercholesterolemic rabbits treated with PCE (HC + PCE), the left atrial enlargement was attenuated (LA/Ao ratio: *p* < 0.0001), the prolongation of the DecT was reduced (*p* < 0.0001), and Tissue Doppler Imaging (TDI) revealed a significant increase in the e’/a’ ratio (*p* = 0.0002) in comparison to the non-treated HC group. The PCE-treated animals exhibited a significant decrease in E/e’ ratio compared to the non-treated rabbits kept on an atherogenic diet (*p* < 0.0001). No significant differences were noted in the results of the Tei-index of the experimental groups (*p* > 0.05). Despite of the long-term hypercholesterolemia, the HC group did not exhibit any indications of deteriorated left ventricle systolic function. Heart rate (HR), ejection fraction (EF), and fractional shortening (FS) remained in the normal range in all cases. Systolic myocardial velocity (s’) and mitral annular plane systolic excursion (MAPSE) values were unaffected by both the atherogenic diet and the anthocyanin administration. The speckle-tracking echocardiographic technique showed a reduced global longitudinal strain (GLS) in the HC group in comparison to the controls, and restored GLS values were observed in the HC + PCE-treated rabbits. Last but not least, no remarkable differences were found in the left ventricle outflow tract (LVOT) parameters, including mean and maximal outflow velocities (Vmax, Vmean) and pressure gradients (maxPG, meanPG).

### 2.4. Anthocyanin-Rich PCE Did Not Improve the Endothelial Dysfunction Induced by Atherogenic Diet

**Figure 3:**
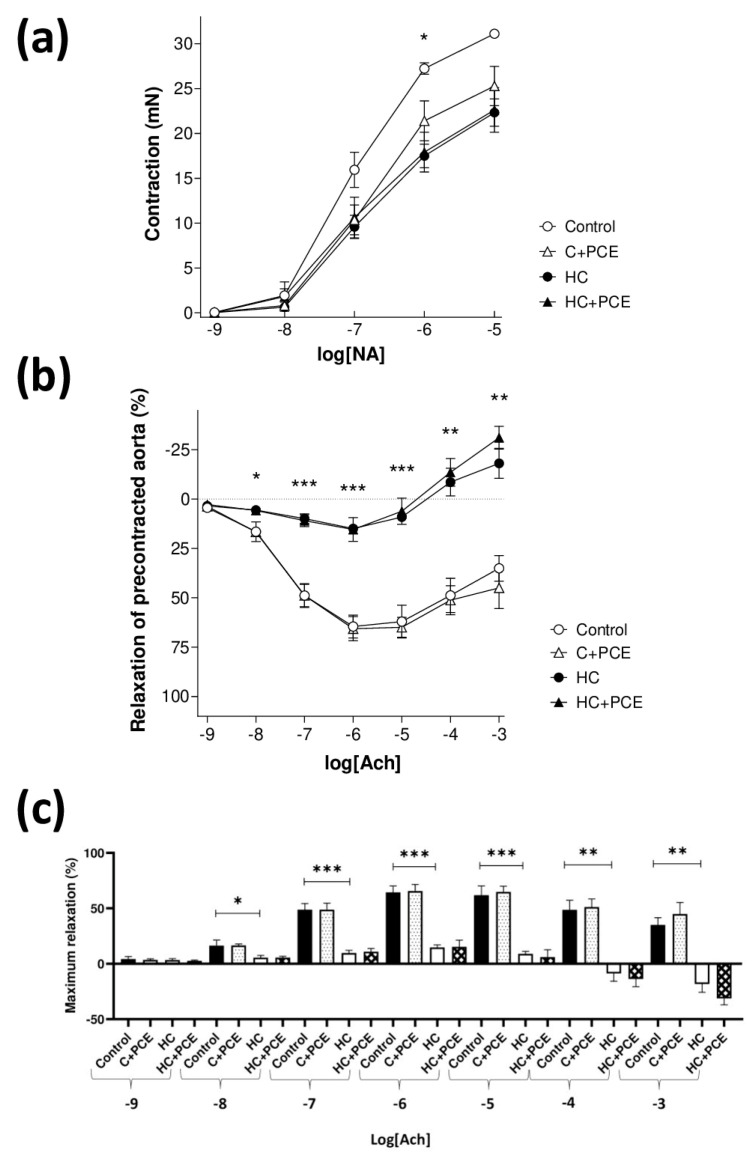
Outcomes of the ex vivo vascular study (*n* = 7/group). The effect of noradrenaline (NA) on panel (**a**) and acetylcholine (Ach) on panel (**b**) on the thoracic aorta isolated from rabbits receiving normal or cholesterol-rich chow with or without an in vivo PCE treatment. The *x* axis shows the common logarithm of molar concentration of noradrenaline (NA) on panel (**a**) and acetylcholine (Ach) on panel (**b**), while the *y* axis indicates the contractile force over the resting level on panel (**a**) and the effect as a percentage decrease in the initial tension of aortic rings on panel (**b**). In the case of acetylcholine (Ach), all aortic rings underwent a previous precontraction evoked by the half-maximal effective concentration (EC_50_) of noradrenaline. The symbols represent the effect of noradrenaline or acetylcholine averaged within the groups (± SEM). On panel (**c**), the maximal relaxation of the groups is presented (mean ± SEM). Asterisks denote the significance level of differences between the control and HC groups (*: *p* < 0.05, **: *p* < 0.01, ***: *p* < 0.001).

The contractions of the aortic rings evoked by noradrenaline (NA) were significantly lower in the HC rabbits in comparison to the controls at 1 µmol/L NA concentration. In all the groups, acetylcholine (Ach), up to 1 µmol/L concentration, elicited relaxation, while higher Ach concentrations caused contraction instead. As expected, the in vivo cholesterol treatment statistically significantly decreased the acetylcholine-evoked relaxation of aortic rings with the preservation of the acetylcholine-evoked contraction (measured in the maximally relaxed state). The in vivo PCE treatment did not influence the vascular response to Ach in a statistically significant manner (Figure 3). Thus, rabbits receiving oral cholesterol treatment developed considerable endothelial dysfunction that could not be treated with simultaneous oral PCE treatment.

### 2.5. Already Existing Atherosclerotic Plaque Coverage Was Not Reduced by the PCE Treatment

**Figure 4:**
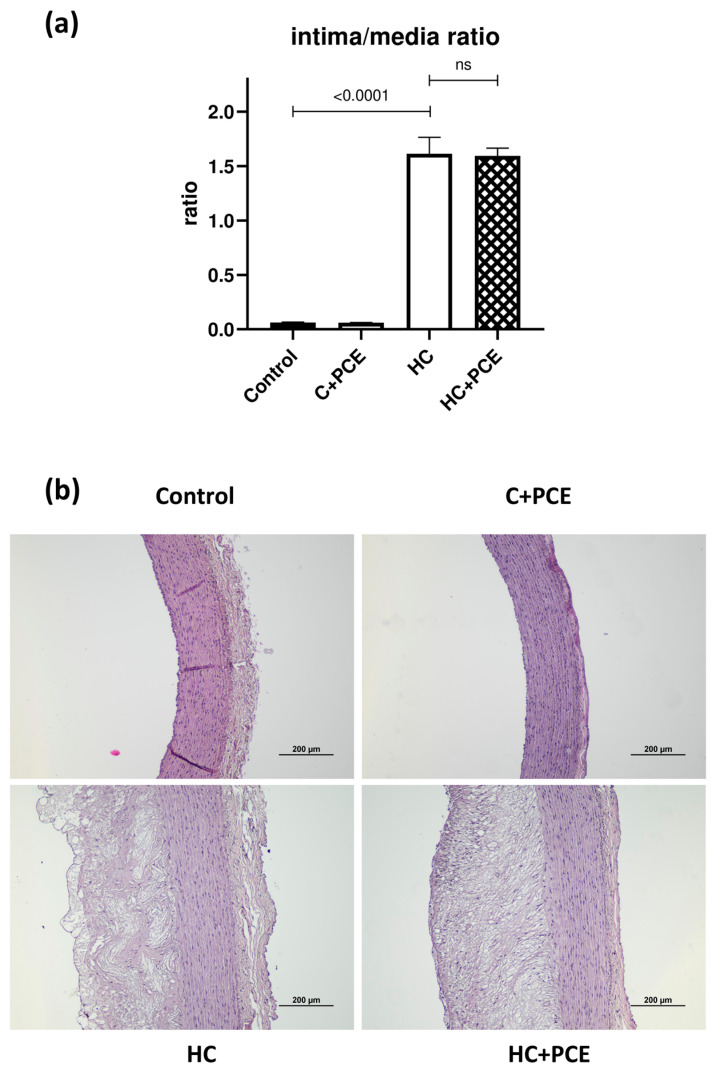
Histological analysis of the aortas of the four experimental groups (*n* = 7 in each). (**a**) Graph shows the averaged intima/media ratios demonstrating a significant increase in the HC animals in comparison to controls (*p* < 0.0001, ns = not significant). Data followed Gaussian distribution. Data were analyzed with ordinary one-way ANOVA and are presented as means ± SEM. (**b**) Representative images of the cross-sectioned aortas (magnification: 10×).

The histological sections of the HE-stained aortas are demonstrated in Figure 4. The samples of both the control and the C + PCE groups were free from atherosclerotic lesions. The thickness of the intimal layer of the animals kept on an atherogenic diet markedly increased because of the excessive foamy plaque coverage. The calculated intima/media ratios were significantly higher in the HC and HC + PCE rabbits in comparison to the healthy controls (*p* < 0.0001) (Figure 4). No significant differences were found between the aortas harvested from the non-treated and PCE-administered HC animals (*p* > 0.05). 

### 2.6. PCE Administration Restored the Myocardial eNOS, PKG, SERCA2a, and HO-1 Levels Altered by the Cholesterol-Rich Diet

**Figure 5:**
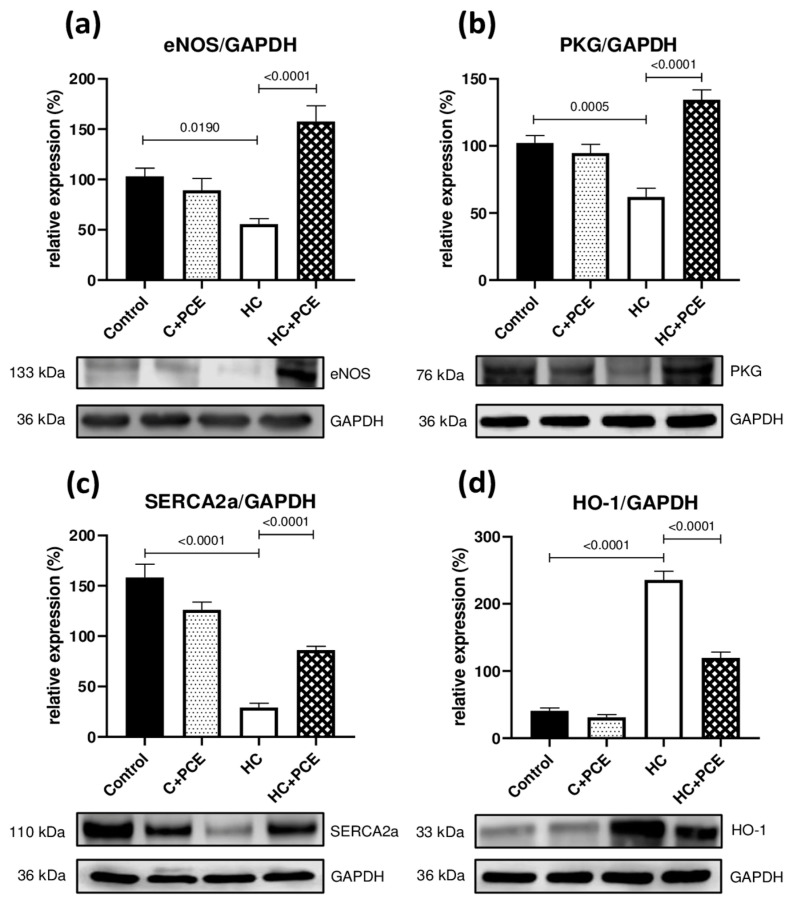
Representative Western blot images of the evaluated proteins, along with quantitative analysis of the density of the bands normalized to GAPDH (*n* = 4 per group). The atherogenic diet significantly reduced the expression of the eNOS (panel (**a**)), PKG (panel (**b**)), and SERCA2a (panel (**c**)) proteins in the LV samples of HC rabbits in comparison to controls. Significantly increased protein levels were detected in hypercholesterolemic rabbits treated with PCE, compared to the non-treated HC group. Hypercholesterolemia-associated increase in HO-1 expression was also observed (**d**); however, PCE treatment reduced the levels of the enzyme in the HC + PCE rabbits. All data are presented as means ± SEM. D’Agostino–Pearson normality test was used to estimate Gaussian distribution and then data were analyzed with ordinary one-way ANOVA.

The left ventricle tissue expression of four decisive proteins of cardiovascular homeostasis is shown in Figure 5. The myocardial eNOS level was significantly lower in HC rabbits compared to the healthy control group (*p* = 0.0190). Similarly, in the case of PKG and SERCA2a levels, significant differences were observed between the control and the non-treated HC groups (PKG: *p* = 0.0005; SERCA2a: *p* < 0.0001). In HC + PCE rabbits, the expression of eNOS, PKG, and SERCA2a proteins was significantly enhanced compared to the group kept only on a cholesterol-rich diet (HC vs. HC + PCE: *p* < 0.0001 for all three proteins). 

Western blot analysis also revealed that the level of HO-1 was significantly higher in the myocardium of rabbits maintained on an atherogenic diet, compared to the expression detected in the healthy control group (*p* < 0.0001). The enhanced HO-1 expression was attenuated by PCE administration, as a significant difference was observed between the HC and HC + PCE groups (*p* < 0.0001). Additionally, for all proteins of interest, no significant differences were observed between the two healthy groups (control vs. C + PCE).

## 3. Discussion

In the present study, we analyzed the possible therapeutic effects of long-term anthocyanin administration in a rabbit model of atherosclerosis-associated cardiac dysfunction.

### 3.1. Diastolic Dysfunction

Our echocardiographic outcomes show that the hypercholesterolemic rabbits suffered from diastolic but not systolic dysfunction. Rabbits fed a cholesterol-rich diet showed no perceptible signs of systolic impairment as ejection fraction (EF), fractional shortening (FS), and systolic myocardial velocity (s’) remained in the normal range. In the HC group, markedly decreased E/A and e’/a’ ratios and increased deceleration time (DecT) and isovolumetric relaxation time (IVRT) were detected, suggesting incomplete left ventricle (LV) relaxation and thus limited LV filling [30]. Moreover, increased LV filling pressure was estimated due to the significant elevation in the E/e’ ratio of the HC animals, providing hard evidence to establish the diagnosis of diastolic dysfunction (DD) [31]. As a response to LV filling pressure elevation, left atrial enlargement (LAE) was also observed in the HC group (as the LA/Ao ratio increased), further supporting the condition of abnormal diastolic function [32]. The anthocyanin treatment improved the diastolic performance, characterized by decreased LA/Ao, e’/a’ and E/e’ ratios and reduced DecT, suggesting the fact that the administered sour cherry extract exerts a potent effect on the myocardium. Interestingly, in our study, no remarkable differences were found in the results of the global myocardial performance defined by the Tei-index. However, Bruch et al. found that the diagnostic value of the Tei-index is limited in CAD patients with impaired diastolic but preserved systolic function [33]. In light of our echocardiographic findings, the administered anthocyanin-rich *Prunus cerasus* extract effectively attenuated the diastolic dysfunction already developed by the atherogenic diet.

### 3.2. Dyslipidemia and Lipid Profile

Serum analyses were conducted to evaluate parameters that may function as diagnostic indicators for estimating the severity of cardiovascular diseases. The results revealed marked dyslipidemia induced by the atherogenic diet, as the atherogenic index and the total cholesterol, LDL-C, and HDL-C levels were significantly elevated in the hypercholesterolemic group, relative to the healthy control rabbits. These outcomes are directly in consonance with previous data gathered by other authors [34]. However, the anthocyanin treatment decreased the atherogenic index, total cholesterol, and LDL-C levels in the HC + PCE group, demonstrating that the extract may have therapeutic effects in the case of already existing dyslipidemia. A similar conclusion was reached in several studies which support our findings of the blood lipid modulatory properties of dietary anthocyanins [24]. Although further investigations are needed, in line with one of the hypothetical effects of the anthocyanins in the presence of hyperlipidemia, it can be speculated that the administered sour cherry extract exerted inhibition on the cholesteryl ester transfer protein (CETP) [35]. CK-MB levels did not differ significantly in the experimental groups, which can be construed as the absence of acute myocardial infarction (AMI).

### 3.3. Endothelial Dysfunction and Atherosclerotic Plaque Coverage

Scientific evidence shows that dietary cholesterol induces dyslipidemia and atherosclerosis, in which oxidative stress and inflammatory processes cause endothelial dysfunction [36]. Under atherosclerotic conditions, the diminished vascular status is characterized by impaired vasorelaxation to acetylcholine (Ach), as it was first demonstrated by Ludmer et al. [37]. Here, the endothelium-dependent vasodilation evoked by Ach markedly was decreased in the HC rabbits, suggesting severe atherosclerosis due to the cholesterol-rich diet. The observed results suggest a decline in endothelial NO synthase (eNOS)-derived NO activity, resulting in disrupted PKG signaling in the surrounding cardiomyocytes [38,39]. Additionally, the PCE treatment failed to recover the vascular function, which was corroborated by the results of the aortic histological study, as the already developed, marked atherosclerotic plaque coverage was not reduced in the PCE-treated HC group. As the administered anthocyanins did not influence the vascular status, we hypothesize that the walls of the vessels were so severely damaged that the PCE could not exert its therapeutic effect. Although further evidence is needed, as the cardiac function improved, our outcomes give the impression of tissue-specific actions of the PCE used in the treatment of already existing cardiac dysfunction together with advanced atherosclerosis.

### 3.4. Upregulation of the NO-cGMP-PKG Pathway Correlates with the Restoration of the Cardiac Function

As the deterioration of the NO-cGMP-PKG pathway is a main pathophysiological mechanism that contributes to diastolic impairment [40], the Western blot technique was conducted to detect the expression of proteins of this cardioprotective pathway. Moreover, Huang et al. demonstrated that alterations in cardiac function generated by hypercholesterolemia were also associated with decreased myocardial SERCA2a expression and activity [41]. The Western blot results revealed that eNOS, PKG, and SERCA2a were expressed at lower levels in the HC group compared to the quantities of these proteins found in the left ventricle samples harvested from the healthy control animals. These findings suggest downregulated NO-cGMP-PKG signaling in the myocardium of the hypercholesterolemic rabbits, further supporting the condition of abnormal diastolic function. However, Western blot analyses showed significantly enhanced cardiac eNOS, PKG, and SERCA2a expression in the HC animals treated with sour cherry extract, which may contribute to the improvement in diastolic performance. The eNOS-inducer role of the anthocyanins was also reported by other authors, confirming our results [42,43]. The authors of the present study assume that the anthocyanins may elicit therapeutic effects by inducing eNOS expression, thus also upregulating the NO-cGMP-PKG pathway in the myocardium. These effects are consistent with an experiment by Quintieri et al., in which anthocyanidins exerted cardioprotection involving the NO-cGMP-PKG pathway against ischemia/reperfusion injury [44]. Another promising finding was that the anthocyanin-dependent diastolic improvement involved enhanced SERCA2a expression, casting a new light on the myocardial effects of the anthocyanins. Together, the present outcomes confirm that the administered anthocyanin-rich *Prunus cerasus* extract attenuated the atherosclerosis-associated diastolic dysfunction through the upregulation of the NO-cGMP-PKG pathway and the downstream signaling target SERCA2a in the myocardial tissue, without affecting the vascular homeostasis.

### 3.5. Heme Oxygenase-1 Overexpression May Act as a Double-Edged Sword

The stress protein heme oxygenase-1 (HO-1, also known as hsp32) plays a prominent role among the defensive systems responsible for restoring the homeostasis of the cells and exerting cytoprotective activities against oxidative stress and inflammation [45]. These favorable effects are mediated by the production of carbon monoxide (CO), the antioxidants biliverdin and bilirubin, and the degradation of an excessive amount of the pro-oxidant heme [46]. As it has been previously reported in the literature, hypercholesterolemia is closely associated with increased myocardial oxidative stress [47], which is related to HO-1 induction [48]. We detected a similar level of induction in the rabbits maintained on a cholesterol-rich diet. Moreover, in the current study, the expression of HO-1 was found to be declining in the left ventricle of the anthocyanin-rich PCE-treated hypercholesterolemic rabbits. This result is contrary to a variety of experimental studies revealing that pharmacological induction of HO-1 by natural compounds such as resveratrol, curcumin, quercetin, etc., exerts potent defensive effects on the cardiovascular system [49]. Our outcomes are supported by an important novelty describing the fact that the putative protective role of this major antioxidant enzyme has become doubtful [50] as myocardial HO-1 overexpression could be either beneficial or harmful depending on the stress context [48]. Allwood et al. demonstrated evidence of the detrimental effects of the overexpressed cardiac HO-1 in the exacerbation of heart failure induced by aging or pressure overload [51].

In the present report, the mechanism behind the HO-1-downregulating effect of the PCE is not entirely clear, but it may be related to complex interactions between the anthocyanins and the cholesterol metabolism. A likely explanation for this outcome is the alleviated myocardial oxidative stress due to the significant cholesterol-lowering effect of the anthocyanins in the PCE-treated hypercholesterolemic rabbits. As HO-1 expression depends on the duration and severity of hypercholesterolemia, its level may be reduced by improving the lipid profile. Alternatively, the anthocyanins may interact with other signaling pathways in the heart that affect HO-1 expression; therefore, further investigations are needed to fully understand the possible underlying mechanisms.

## 4. Materials and Methods

### 4.1. Anthocyanins

The anthocyanin-rich extract was made from a Hungarian sour cherry (*Prunus cerasus* L.) cultivar called "Érdi bőtermő". The fruit samples were harvested from the orchard of the Research and Consulting Institute for Fruitgrowing (Újfehértó) between June and July of 2020. After the sampling, the fruits were immediately transferred to the laboratory and stored at -20 °C until use.

The procedure for the anthocyanin preparation was carried out at the Institute of Food Technology, University of Debrecen, as described previously [52]. In brief, the sour cherry was thawed, deseeded, and homogenized. Extraction was performed with an ethanol/water/acetic acid mixture. The extract was mixed, filtered, and centrifuged. The anthocyanin-rich supernatant was purified with pre-conditioned Supelclean ENVI-18 SPE (Sigma-Aldrich-Merck KGaA, Darmstadt, Germany) tubes. The anthocyanins were eluted with ethanol containing 20% distilled water, and this fraction was evaporated at 40 °C with a rotary evaporator. The anthocyanin-rich extract was analyzed with a CromasterUltraRs UHPLC (Hitachi High-Tech Corporation, Tokio, Japan) equipped with a diode array detector and OpenLAB software (version A.04.10, Agilent Technologies, Santa Clara, CA, USA), according to a previously defined method [53].

### 4.2. Animals

The present study was carried out using 3-year-old male New Zealand White (NZW) rabbits (Jurasko Ltd, Debrecen, Hungary) with a body weight range of 2500–3000 g. The animals received humane care in accordance with the "Principles of Laboratory Animal Care" by EU Directive 2010/63/EU and all aspects of the experiments were approved by the University of Debrecen Committee of Animal Welfare (4/2022DEMÁB). The animals were kept in individual cages under standard conditions (a 12:12 h dark/light cycle at a temperature of 24 °C). During a 2-week-long adaptation period, the rabbits were fed normal chow and had free access to tap water.

### 4.3. Study Design

At the beginning of the study, the experimental animals were randomly divided into two groups: (1) a healthy control group which received normal rabbit chow and (2) a hypercholesterolemic (HC) group in which rabbits were kept on intermittent cycles of an “atherogenic” diet with periods of a normal diet for 32 weeks. The “atherogenic” chow contained 1% cholesterol and 1% saturated fat and was formulated by Pro Drug L.P. (Debrecen, Hungary) [54,55].

**Figure 6:**
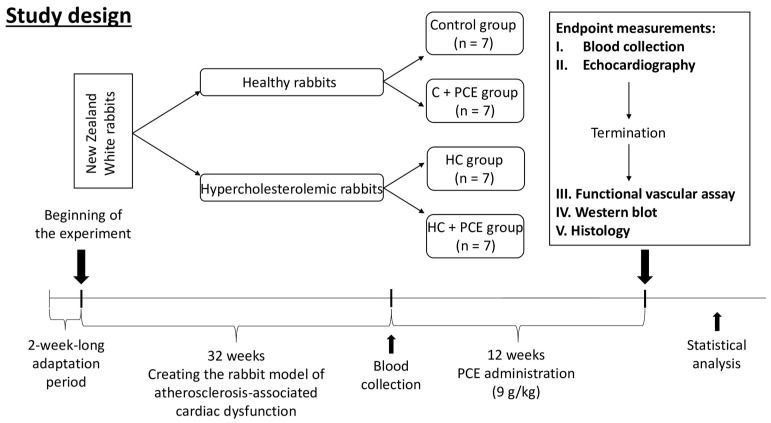
Flowchart of the study. After the acclimatization, half of the rabbits were maintained on normal chow, and half of the rabbits were kept on intermittent cycles of atherogenic diet for 32 weeks. Then, the animals were divided into 4 subgroups (*n* = 7 in each): (1) untreated control group; (2) healthy rabbits treated with 9 g/kg PCE; (3) hypercholesterolemic (HC) animals kept on cholesterol-rich diet; and (4) HC rabbits with 9 g/kg PCE treatment, for 12 weeks. Before and after the administration of the PCE, serum parameters were measured. At the endpoint, cardiac ultrasound was carried out, and after thoracotomy, ex vivo vascular assay and Western blot and histological analyses were performed.

After 32 weeks of creating the rabbit model of atherosclerotic cardiovascular disease, we made four subgroups from the two original ones described previously and then started the administration of the anthocyanin-rich *Prunus cerasus* extract (PCE, 9 g/kg) for 12 weeks [56,57], every single day (Figure 6). Thus, the final experimental groups (*n* = 7 in each) in the study were as follows: (1) the untreated control group received normal chow; (2) the healthy control with PCE treatment (C + PCE) received normal chow and 9 g/kg PCE dissolved in tap water; (3) the hypercholesterolemic (HC) group was kept on atherogenic chow; and (4) the PCE-treated diseased group (HC + PCE) received atherogenic chow and 9 g/kg PCE dissolved in the drinking water. The control and the HC groups were treated with vehicle. The weight of the animals was recorded once a week, and for the two groups drinking anthocyanin solution (C + PCE and HC + PCE), it was freshly prepared every day. A total of 9 g of the PCE contained 100 mg anthocyanins [58] and the required amount of the sour cherry extract (9 g × body mass of the rabbit) was dissolved in 200 mL of tap water and fed to the animals in the early evening hours. The next morning, just after all the solution had been consumed, the drinking bottles were refilled with fresh water.

Before the administration of the PCE, the assessment of the lipid profile of each rabbit was performed. At the endpoint, blood sample collection and echocardiographic measurements were carried out. The animals were sacrificed under deep anesthesia (ketamine/xylazine 150/10 mg/kg, i.m. injection). After thoracotomy, the distal part of the thoracic aorta was excised to perform ex vivo vascular assays. The cardiac samples and the remaining part of the thoracic aorta were immediately frozen in liquid nitrogen and stored at −80 °C for Western blot analysis or in a 10% formalin solution for histology.

### 4.4. Serum Parameters

Before and after the 12-week-long treatment, blood samples were collected from the marginal ear veins of each rabbit into BD Vacutainer SST II Advance Tubes (BD Vacutainer, Bergen County, NJ, USA), after 12 h fasting. The serum levels of total cholesterol, low-density lipoprotein cholesterol (LDL-C), high-density lipoprotein cholesterol (HDL-C), triglyceride (TG), glucose, aspartate transaminase (AST), and alanine transaminase (ALT) were determined. Specific markers like C-reactive protein (CRP), creatine kinase MB isoform (CK-MB), and N-terminal pro-B-type natriuretic peptide (NT-pro-BNP) were measured, and the atherogenic index of plasma (AIP = total cholesterol/HDL-C) was calculated as well. All parameters were recorded in the Department of Laboratory Medicine at the University of Debrecen by using automated clinical laboratory analyzers (Roche Diagnostics GmbH, Mannheim, Germany).

### 4.5. Transthoracic Echocardiographic Technique

**Figure 7:**
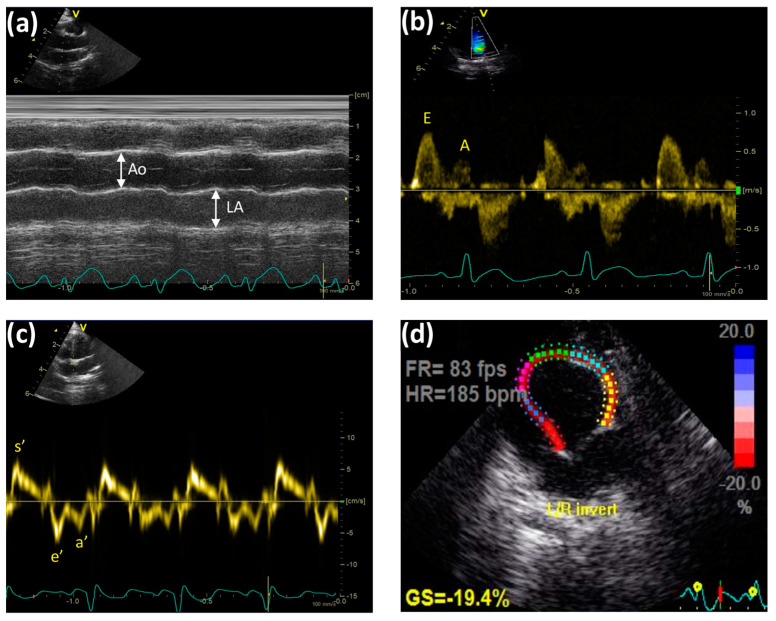
Representative echocardiographic images of the experimental animals. (**a**) M-mode: Ao (aortic root) and LA (left atrium); (**b**) Doppler mode: E (early) and A (atrial) transmitral flow velocities; (**c**) Tissue Doppler Imaging (TDI) technique: s’ (systolic myocardial velocity), e’ (early), and a’ (atrial) diastolic myocardial velocities; (**d**) speckle tracking modality (FR: frame rate; HR: heart rate; GS: global strain, L/R invert: left/right invert).

At the endpoint, cardiac ultrasound was carried out using a Vivid E9 ultrasound machine (GE Healthcare, New York, NY, USA) with a high-frequency probe (12S-D) (Figure 7). Rabbits were under light anesthesia provided by a mixture of ketamine/xylazine (35/3 mg/kg, intramuscular injection). After shaving the chests of the animals and positioning them into dorsal and lateral decubitus positions, echocardiographic measurements were performed as recommended by the American Society of Echocardiography [59]. Echocardiograms were recorded from the parasternal long axis (PLAX), parasternal short axis (PSAX), and also from apical 4- and 5-chamber views. Two-dimensional B-mode, M-mode, pulsed-wave (PW) Doppler, and Tissue Doppler Imaging (TDI) echocardiographic techniques were carried out, with continuous ECG monitoring. M-mode traces were recorded to determine the diameter of the aortic root (Ao, mm), the left atria (LA, mm), and at the mid-papillary muscle level to measure the ejection fraction (EF, %), the fractional shortening (FS, %), the end-diastolic and end-systolic LV diameters (EDD and ESD, respectively; mm), and the anterior and posterior wall thickness of the LV in systole and diastole (LVAWs,d; LVPWs,d, respectively; mm). The E (early) and A (atrial) transmitral flow velocities (mm/s), E/A ratio, and deceleration time of the E wave (DecT, ms) were assessed using the Doppler technique to estimate the diastolic function of the LV. From the apical five-chamber view, left ventricle outflow tract (LVOT) pressure gradients (PG, mmHg) and velocities (V, mm/s) were evaluated. TDI measurements were conducted to define the motion of the myocardium: systolic myocardial velocity (s’, mm/s) and early (e’, mm/s) and atrial (a’, mm/s) diastolic myocardial velocities were recorded at the septal and lateral walls of the mitral annulus; thus, the e’/a’ ratio was calculated. Mitral valve closure-to-opening time (MCOT, ms), ejection time (ET, ms), isovolumetric contraction time (IVCT, ms), and isovolumetric relaxation time (IVRT, ms) were measured from the TDI recordings. Myocardial Performance Index (MPI, Tei-index) was defined as IVCT + IVRT/ET, and the E/e‘ ratio (indicative for LV filling pressure) was also calculated. The traces were stored on an external hard drive and later analyzed by a blinded expert using EchoPAC PC software (ver. 112, GE Healthcare, New York, NY, USA). Using the apical long axis (APLAX) and 4-chamber-view recordings, the speckle-tracking technique was used to analyze myocardial strain using the Q-analysis/2D Strain tool of the EchoPAC PC software. The region of interest (ROI) representing the endocardial wall was defined manually, and the Global Longitudinal Strain (GLS, %) was automatically calculated.

### 4.6. Functional Vascular Assays

After sacrificing the rabbits, the distal part of the thoracic aorta was isolated, and 2 mm wide rings were cut off (four rings from each animal). The rings were mounted horizontally at a 10 mN resting tension using a wire instrument (Experimetria Ltd., Budapest, Hungary) in a 4-chamber isolated organ bath system with 10 mL vertical chambers (TSZ-04; Experimetria Ltd., Budapest, Hungary). The chambers were filled with Krebs solution oxygenated with 95% O_2_ and 5% CO_2_ (36 °C; pH = 7.4). The Krebs solution contained (in mmol/L): NaCl: 118, KCl: 4.7, CaCl_2_: 2.5, NaH_2_PO_4_: 1, MgCl_2_: 1.2, NaHCO_3_: 24.9, glucose: 11.5, and ascorbic acid: 0.1, dissolved in redistilled water. The isometric contractile force of the circulatory muscle layer was measured using a transducer (SD-01; Experimetria Ltd., Budapest, Hungary) connected to a WS-DA-02 workstation (MDE Research) with SPEL Advanced Isosys software version 2.9 (SOFT-02; MDE Research, Heidelberg, Germany).

After a 60 min incubation period, a noradrenaline (NA) concentration–response (E/c) curve (from 1 nmol/L to 10 µmol/L) was generated, and the half-maximal effective noradrenaline concentration (EC_50_) was determined for each aortic ring. After a 60 min wash-out, the EC_50_ of noradrenaline was administered to each ring and, after the stabilization of the contractile force, an acetylcholine (Ach) E/c curve was constructed (from 1 nmol/L to 1 mmol/L).

The responses of aortic rings obtained from the same animal were averaged. The effect of noradrenaline was defined as the maximal phasic (quick) increase in the vascular tension beyond the resting level (adjusted previously to 10 mN). In turn, the effect of acetylcholine was defined as the percentage decrease in the vascular tonic (stabilized) tension produced by the EC_50_ of noradrenaline. If acetylcholine evoked relaxation, maximal relaxation was considered. If contraction occurred, its maximum was taken into account (as negative relaxation).

### 4.7. Western Blot

The Western blot technique was used to identify proteins from the left ventricle (LV). Firstly, for protein isolation, 300 mg of deep-frozen LV tissue samples were homogenized in 800 µl buffer solution (25 mM Tris, 25 mM NaCl, 1 mM Na-orthovanadate, 10 mM NaF, 10 mM Na-pyrophosphate, 10 nM okadaic acid, 0.5 mM EDTA, 1 mM PMSF, protease inhibitor cocktail, and distilled water (all from Sigma-Aldrich-Merck KGaA, Darmstadt, Germany)), using a disperser (IKA-WERKE, Staufen, Germany). Then, the homogenates were centrifuged at 2000 rpm for 10 min at 4 °C. The supernatant contained the cytosolic and mitochondrial fractions. All samples were incubated on ice for 1 h in a solution containing 0.1% Triton-X-100 (Sigma-Aldrich-Merck KGaA, Darmstadt, Germany). After a second centrifugation at 14000 rpm for 20 min at 4 °C, the resulting supernatants were used as the cytosolic–mitochondrial extracts. From 10 μL of the samples, the total protein concentration was measured with the help of a spectrophotometer (FLUOstar Optima, BMG Labtech, Ortenberg, Germany), using the BCA assay (QuantiPro BCA Assay Kit, Sigma-Aldrich-Merk KGaA, Darmstadt, Germany). Next, 50 μL of the residual volume was diluted with Laemmli buffer (Sigma-Aldrich-Merck KGaA, Darmstadt, Germany), and finally, the remaining samples were stored at −80 °C.

Samples mixed with Laemmli buffer were separated via SDS–polyacrylamide gel electrophoresis (SDS-PAGE) (12% gel, 25 mA) in a running buffer, and then proteins were transferred onto a nitrocellulose membrane (GE Healthcare, Darmstadt, Germany) in a transfer buffer at 25 V for 90 min. After 1 h of blocking with 5% BSA solution (Sigma-Aldrich-Merck KGaA, Darmstadt, Germany), blots were incubated overnight at 4 °C with primary antibodies: anti-glyceraldehyde-3-phosphate-dehydrogenase (GAPDH, as a housekeeping protein; Cat.No: G8795, Sigma-Aldrich-Merck KGaA, Darmstadt, Germany); anti-endothelial nitric oxide synthase (eNOS, Cat.No: ab5589, Abcam Plc., Cambridge, UK); anti-protein kinase G (PKG, Cat.No: ab97339, Abcam Plc., Cambridge, UK); anti-sarco/endoplasmic reticulum calcium ATPase 2a (SERCA2a, Cat.No: ab2817, Abcam Plc., Cambridge, UK); and anti-heme oxygenase-1 (HO-1, Cat.No: SAB2108676, Sigma-Aldrich-Merck KGaA, Darmstadt, Germany). The following morning, the membranes were washed with TBS-T for 3 × 10 min before being incubated with horseradish–peroxidase (HRP)-conjugated secondary (anti-mouse or anti-rabbit) antibodies. To identify and detect protein bands, enhanced chemiluminescent substrate (WesternBright™, ECL, Advansta Inc., San Jose, CA, USA) and LiCor C-Digit^®^ blot scanner (LI-COR Inc., Lincoln, NE, USA) were used. The analysis of the scanned Western blots was carried out using Image Studio Digits ver. 5.2. software (LI-COR Inc., Lincoln, NE, USA). In all cases, the normalization to the background and the standardization to a housekeeping protein (GAPDH) were conducted. Finally, data analysis was performed on three independent experiments.

### 4.8. Histology

To visualize tissue structure, aortic samples were fixed in 10% neutral buffered formalin (pH = 7.4) for 24 h. The following day, tissue samples were washed in water for 1 h and then stored in 70% ethanol until further steps. After dehydration and clearing with xylene, from the final paraffin-embedded block 5 μm thick slices were sectioned, deparaffined, and stained with hematoxylin–eosin (HE). For image acquisition, a Nikon Eclipse 80i microscope (Nikon Corp., Tokio, Japan) was used. From the cross-sectioned aortic samples the thickness of the intima and the media was measured using the length-measuring function of the Nikon NIS-Elements BR (Ver5.41.00) software (Nikon Corp., Tokio, Japan), followed by the calculation of the average intima/media ratios. 

### 4.9. Statistical Analysis

Data analysis was performed using GraphPad Prism software for Windows, version 8.00 (GraphPad Software Inc., La Jolla, CA, USA). The D’Agostino–Pearson test was used first to determine the Gaussian distribution. According to the result of the normality test, further statistical analysis was carried out using either one-way analysis of variance (ANOVA) followed by Tukey’s post hoc analysis or (if the normality test was not passed) the Kruskal–Wallis test followed by Dunn’s post-test. Results were considered to be significantly different when the probability values (*p*) were less than 0.05 (*p* < 0.05). All data are expressed as means ± standard error of the means (SEM).

## 5. Conclusions

The primary outcome of the recent study is that the upregulation of the cardiac NO-PKG pathway along with SERCA2a induction as a result of a 12-week-long treatment with *Prunus cerasus* extract (PCE) attenuated the already existing cardiac diastolic dysfunction induced by an atherogenic diet without influencing the vascular status. This experiment also adds to the growing body of research showing that HO-1 overexpression may act as a double-edged sword in the maintenance of cell homeostasis. PCE administration restored the diastolic dysfunction observed in the untreated hypercholesterolemic animals and induced the upregulation of the cardioprotective eNOS, PKG, and SERCA2a proteins. Taking into consideration the positive results of the PCE treatment, incorporating dietary anthocyanin supplementation could offer a promising therapeutic approach in the treatment of cardiac dysfunction linked to atherosclerosis.

## Figures and Tables

**Table 1:** Endpoint serum parameters (*n* = 7 in each group).

Serum Parameter	Control	C + PCE	HC	HC + PCE
Total cholesterol (mmol/L)	0.72 ± 0.083	0.478 ± 0.066	34.79 ± 1.319 ****	25.02 ± 2.546 ^##^****
LDL-C (mmol/L)	0.096 ± 0.007	0.071 ± 0.012	32.99 ±0.694 ****	21.80 ± 2.054 ^####^****
HDL-C (mmol/L)	0.452 ± 0.049	0.282 ± 0.060	1.714 ± 0.408 **	1.458 ± 0.197 **
Atherogenic index of plasma (AIP) (TC/HDL)	1.613 ± 0.075	2.102 ± 0.313	28.17 ± 4.225 ****	18.09 ± 0.756 ^##^****
Triglyceride (mmol/L)	0.968 ± 0.227	0.829 ± 0.157	1.239 ± 0.195	1.635 ± 0.368
CRP (mg/L)	0.2 ± 0.104	0.241 ± 0.091	0.815 ± 0.649	0.15 ± 0.023
CK-MB (U/L)	217.7 ± 58.62	446.0 ± 125.1	477.4 ± 75.19	480.9 ± 82.50
AST (GOT) (U/L).	20.60 ± 2.768	20.78 ± 2.159	30.75 ± 6.421	30.22 ± 4.300
ALT (GPT) (U/L)	56.76 ± 7.115	44.62 ± 3.042	37.98 ± 7.127	43.01 ± 7.825
Glucose (mmol/L)	5.473 ± 0.217	6.774 ± 0.348	6.940 ± 0.552	7.040 ± 0.255

The atherogenic diet significantly increased the serum lipid parameters in the HC and HC + PCE groups; however, the PCE treatment reduced both the TC and the LDL-C levels and decreased the AIP compared to the HC animals. No significant changes were observed in the CRP, CK-MB, glucose, AST, and ALT levels in the experimental groups. To estimate Gaussian distribution, D’Agostino–Pearson normality test was used; then, the data were analyzed with ordinary one-way ANOVA or Kruskal–Wallis test. All data are presented as means ± SEM (** *p* < 0.01, **** *p* < 0.0001 compared to controls; ^##^ *p* < 0.01, ^####^ *p* < 0.0001 compared to the HC group). LDL-C: low-density lipoprotein cholesterol; HDL-C: high-density lipoprotein cholesterol; AIP: atherogenic index of plasma; CRP: C-reactive protein; CK-MB: creatine kinase MB isoform; AST: aspartate transaminase; ALT: alanine transaminase.

**Table 2:** Systolic and LVOT parameters (*n* = 7 per group).

Parameter	Control	C + PCE	HC	HC + PCE
Tei-index	0.7939 ± 0.044	0.7792 ± 0.021	0.7670 ± 0.050	0.7637 ± 0.027
HR (bpm)	174.4 ± 5.066	195.5 ± 5.726	153.4 ± 8.394	158.3 ± 4.040
EF (%)	65.09 ± 3.497	71.31 ± 2.690	63.38 ± 4.946	72.52 ± 1.896
FS (%)	33.55 ± 2.602	38.23 ± 2.107	32.88 ± 3.351	39.81 ± 1.677
s’ (mm/s)	71.30 ± 4.858	77.62 ± 2.973	61.88 ± 4.951	70.86 ± 3.621
MAPSE (mm)	4.628 ± 0.175	4.507 ± 0.229	3.990 ± 0.289	4.955 ± 0.268
LVOT Vmax (m/s)	1.027 ± 0.047	1.133 ± 0.046	1.119 ± 0.067	1.125 ± 0.039
LVOT Vmean (m/s)	0.656 ± 0.027	0.725 ± 0.035	0.71 ± 0.036	0.69 ± 0.026
LVOT maxPG (mmHg)	4.284 ± 0.385	5.219 ± 0.423	5.109 ± 0.615	5.185 ± 0.378
LVOT meanPG (mmHg)	2.110 ± 0.180	2.417 ± 0.278	2.566 ± 0.264	2.453 ± 0.186

No significant changes were noted between the results of the Tei-index, systolic function, and LVOT parameters. D’Agostino-Pearson test was used to estimate Gaussian distribution, then data were analyzed with ordinary one-way ANOVA or Kruskal–Wallis test. All data are presented as means ± SEM. HR: heart rate; EF: ejection fraction; FS: fractional shortening; s’: systolic myocardial velocity; MAPSE: mitral annular plane systolic excursion; LVOT: left ventricle outflow tract; Vmax: maximal velocity, Vmean: mean velocity; maxPG: maximal pressure gradient; meanPG: mean pressure gradient.

## Data Availability

The data that support the findings of this study are available from the corresponding author upon reasonable request.

## Abbreviations

A: atrial wave of the transmitral flow; a’: atrial diastolic myocardial velocity; Ach: acetylcholine; ACN: acetonitrile; AIP: atherogenic index of plasma; ALT: alanine transaminase; AMI: acute myocardial infarction; Ao: aortic; APLAX: apical long axis; AST: aspartate transaminase; Ca^2+^: calcium; CAD: coronary artery disease; CETP: cholesteryl ester transfer protein; cGMP: cyclic guanosine monophosphate; CK-MB: creatine kinase MB isoform; CO: carbon monoxide; CRP: C-reactive protein; DD: diastolic dysfunction; DecT: deceleration time; E: early wave of the transmitral flow; e’: early diastolic myocardial velocity; EC_50_: half-maximal effective concentration; ECG: electrocardiogram; ECM: extracellular matrix; EDD: end-diastolic diameter; EF: ejection fraction; eNOS: endothelial nitric oxide synthase; ESD: end-systolic diameter; ET: ejection time; FS: fractional shortening; GAPDH: glyceraldehyde-3-phosphate-dehydrogenase; GLS: Global Longitudinal Strain; HC: hypercholesterolemic; HDL-C: high-density lipoprotein cholesterol; HE: hematoxylin–eosin; HF: heart failure; HO-1: heme oxygenase-1; HR: heart rate; HRP: horseradish–peroxidase; hsp: heat shock protein; i.m.: intramuscular; IVCT: isovolumetric contraction time; IVRT: isovolumic relaxation time; LA: left atria; LAE: left atrial enlargement; LDL-C: low-density lipoprotein cholesterol; LV: left ventricle; LVAW: left ventricle anterior wall; LVOT: left ventricular outflow tract; LVPW: left ventricular posterior wall; MAPSE: mitral annular plane systolic excursion; maxPG: maximal pressure gradient; MCOT: mitral valve closure to opening time; meanPG: mean pressure gradient; MPI: Myocardial Performance Index; NA: noradrenaline; NO: nitric oxide; NT-pro-BNP: N-terminal pro-B-type natriuretic peptide; NZW: New Zealand White; PCE: *Prunus cerasus* extract; PKG: protein kinase G; PLAX: parasternal long axis; PSAX: parasternal short axis; PW: pulsed wave; ROI: region of interest; s’: systolic myocardial velocity; SERCA2a: sarco/endoplasmic reticulum Ca^2+^-ATPase; sGC: soluble guanylate cyclase; TDI: Tissue Doppler Imaging; TG: triglyceride; UHPLC: Ultra-High-Performance Liquid Chromatography; Vmax: maximal velocity; Vmean: mean velocity.
